# Epidemiological Characteristics of Mumps Under Different Immunization Strategies in Henan Province

**DOI:** 10.3390/vaccines14010100

**Published:** 2026-01-20

**Authors:** Zhanpei Xiao, Mingxia Lu, Yating Ma, Yan Wang, Mingyu Zhang, Binghui Du, Yiran Bai, Yuzhu Ma, Yanyang Zhang

**Affiliations:** Henan Provincial Center for Disease Control and Prevention, Zhengzhou 450016, China; xiaozp@hncdc.com.cn (Z.X.); lumx@hncdc.com.cn (M.L.); mayt@hncdc.com.cn (Y.M.); wangy@hncdc.com.cn (Y.W.); zhangmy@hncdc.com.cn (M.Z.); dubh@hncdc.com.cn (B.D.); baiyr@hncdc.com.cn (Y.B.); mayz@hncdc.com.cn (Y.M.)

**Keywords:** mumps, mumps-containing vaccine coverage, immunization strategy, epidemiological characteristics

## Abstract

Background: On 1 January 2019, a 2-dose mumps-containing vaccine (MuCV) immunization strategy was adopted in Henan Province before the national Expanded Program on Immunization (EPI). This study examines the epidemiological characteristics of mumps cases during the implementation of various immunization strategies in Henan Province. Methods: We employed descriptive statistics and initially retrieved data on reported cases from 2004 to 2024. Mumps case data were sourced from the China Information System for Disease Control and Prevention (CISDCP). Results: Between 2004 and 2024, a total of 301,342 cases of mumps disease were reported in Henan Province, and the average annual reported incidence was 15.11 cases per 100,000 people. The average yearly incidence decreased by 60.29% following the implementation of the 2-dose vaccination strategy compared with the one-dose strategy. The study identifies two annual incidence peaks from 2004 to 2024: a prominent peak from April to July and a smaller peak from December to January. From 2019 to 2024, in addition to a slight dip in February, the seasonality was less pronounced, with cases distributed sporadically throughout the year. The proportion of the population over 20 years old increased annually, from 8.17% in 2004–2008 to 15.55% in 2019–2024. There was an overall negative correlation between the estimated MuCV vaccination rate and the reported incidence of mumps (r = −0.685, *p* < 0.05). Conclusions: The introduction of 2-dose MuCV in the EPI significantly reduced the incidence rate and total number of cases. While continuing the 2-dose MuCV immunization strategy in the future, it is crucial to remain vigilant in preventing and controlling mumps among individuals over 20 years old.

## 1. Introduction

Mumps is a common childhood infection caused by the mumps virus (R_0_ = 10) [1] and is characterized clinically by nonsuppurative inflammation of the parotid gland, along with swelling and pain in the parotid area. Although typically mild, the disease can lead to serious complications, including orchitis, oophoritis, pancreatitis, encephalitis, meningitis, and deafness [2]. Approximately 30% of postpubertal male patients with mumps orchitis suffer from infertility or subfertility [3]. Previous studies have shown that mumps is a major cause of male infertility and acquired deafness in children [4]. The prevalence of mumps cases in China has consistently increased annually, positioning it as the second most prevalent class C infectious disease after influenza between 2016 and 2019 [5].

Vaccination with the mumps-containing vaccine (MuCV) is the most cost-effective and efficient measure for the prevention and control of mumps [6]. The World Health Organization reported that in countries with a high coverage rate of two doses of MuCV, the incidence of mumps decreased by more than 95% compared with that in the prevaccine era. Since the 1990s, China has provided preventive vaccination services with MuCV on a voluntary and self-paid basis. Since 2008, the Expanded Program on Immunization (EPI) has offered a complimentary single-dose combined vaccine targeting measles, mumps, and rubella (MMR) to children within the age range of 18 to 24 months [7]. In June 2020, immunization programs involving 2 doses of MMR were given at the ages of 8 and 18 months [5].

Henan Province is located in the lower reaches of the Yellow River and in the central-eastern part of China; it covers a total area of 167,000 square kilometres and has a total population of approximately 98 million in 2024. The province is divided into 18 administrative regions. Most of the province lies in the warm temperate zone, and there are approximately 3800 vaccination units in the whole province. In 2009, Henan Province fully implemented an immunization strategy involving one dose of MuCV in accordance with the EPI requirements. On 1 January 2019, Henan Province implemented a 2-dose MuCV immunization strategy earlier than the national EPI.

There are significant differences in the protective effects of MuCV under different immunization strategies [8]. This study describes the epidemiological changes caused by MuCV before and after the implementation of an updated vaccination schedule in Henan Province and to evaluate the prevention and control effects of mumps under different immunization strategies.

## 2. Materials and Methods

### 2.1. Data Resources and Target Population

All instances of mumps and measles are sourced from the China Information System for Disease Control and Prevention (CISDCP). The case types are clinically diagnosed and confirmed. According to the onset dates, the dates of mumps cases were from 1 January 2004 to 31 December 2024, and those of measles cases were from 1 January 2009 to 31 December 2024. The data on cases was retrieved from CISDCP on 30 July 2025. The data included the patient’s name, gender, date of birth, onset date, age, address, and history of MMR vaccination.

### 2.2. Estimation of MuCV Coverage

Since all MuCVs are the measles–mumps–rubella vaccines (MMR) in Henan Province, the vaccination rate of measles-containing vaccines (MCVs) is equal to that of MuCVs. Therefore, in this study, the MuCV vaccination rate was estimated by determining the MCV vaccination rate. The formula for the vaccination rate of the MCV recommended by the WHO is as follows: PPV = PCV/[1 − (1 − PCV) × VE], where PPV is the estimated vaccination rate of the MCV, PCV is the proportion of measles patients with a history of vaccination (those with an unknown immunization history are regarded as unvaccinated), and VE represents vaccine efficacy, with a theoretical value of 95% [9].

### 2.3. Division of Immunization Strategy Periods

Based on the different doses of MuCV in Henan Province, the period of this study was divided into three periods: 2004–2008, the period before the government implemented free MuCV; 2009–2018, the period of the one-dose vaccination schedule; and 2019–2024, the period of the two-dose vaccination schedule.

### 2.4. Descriptive Statistical Analysis

Data processing and statistical analyses were performed using Microsoft Office Excel (version 2016) and SPSS 23.0. Descriptive statistics summarize incidence by person, place, and time. Chi-square tests (Pearson chi-square where applicable) compared incidence rates and vaccine coverage. The incidence rates were calculated for each region and time period, with the corresponding 95% confidence intervals (95% CI) provided. The primary focus was on changes in morbidity and vaccine coverage before and after inclusion of MuCV in the EPI and before and after introduction of MuCV-1 and MuCV-2. A *p* value < 0.05 was considered statistically significant.

## 3. Results

### 3.1. General Characteristics

From 2004 to 2024, a total of 301,342 cases of mumps disease were documented in Henan Province, and the average annual reported incidence was 15.11 cases per 100,000 people. The greatest reported incidence of mumps occurred in 2009–2018, with a rate of 20.08 cases per 100,000 people (Table 1). The mumps incidence in 2019–2024 was significantly different from that in 2004–2008 (χ^2^ = 8527.617, *p* < 0.05)and 2009–2018 (χ^2^ = 34,749.354, *p* < 0.05), which was caused by the inclusion of the two-dose MuCV in the EPI in Henan Province in 2019.

### 3.2. Seasonal Distribution

**Figure 1:**
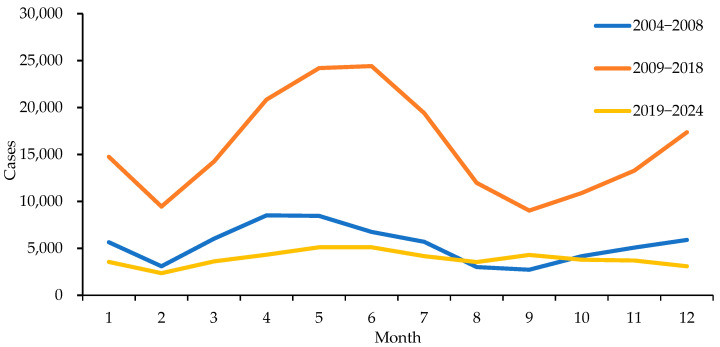
Incidence of mumps by month in Henan Province, 2004–2024.

Mumps cases were reported in all months between 2004 and 2024. From 2004 to 2018 in Henan Province, the incidence of mumps was seasonal with two annual peaks of mumps incidence; a prominent peak from April to July and a small peak from December to January. The number of cases during the peak periods (161,890 cases) accounted for 63.54% of the total reported cases (254,780 cases). From 2019 to 2024, except for a slightly lower number of cases in February, the seasonality was not as obvious, and the cases were sporadically distributed throughout the year (Figure 1).

### 3.3. Geographic Distribution

Table 2 shows the average annual incidence rates for each district in Henan Province from 2004 to 2024. The differences in incidence in Henan Province before and after the inclusion of the 1-dose MuCV vaccine in EPI were statistically significant, and the incidence was compared across regions in 2008–2018 and 2019–2024.

From 2004 to 2024, the top three average annual incidences in Henan Province occurred in Jiyuan (25.86 per 100,000), Zhengzhou (23.80 per 100,000) and Anyang District (22.79 per 100,000). The highest incidences in 2004–2008, 2009–2018, and 2019–2024 occurred in Hebi, Jiyuan and Jiyuan, respectively.

Following the implementation of the two-dose vaccination regimen, cases per district declined by approximately 52.90% relative to the one-dose schedule. The largest reductions occurred in Xinxiang (73.95%), Pingdingshan (73.68%), and Nanyang (66.33%).

### 3.4. Gender Distribution

There were 192,326 male cases and 109,016 female cases among the reported cases of mumps from 2004 to 2024 in Henan Province, with a male-to-female ratio of 1.76:1. The male-to-female ratios in 2004–2008, 2009–2018, and 2019–2024 were 1.84:1, 1.80:1, and 1.43:1, respectively. The average incidence rate of males (18.88 per 100,000) was significantly greater than that of females (11.17 per 100,000) (χ^2^ = 19,579.909, *p* < 0.05).

### 3.5. Age Distribution

**Figure 2:**
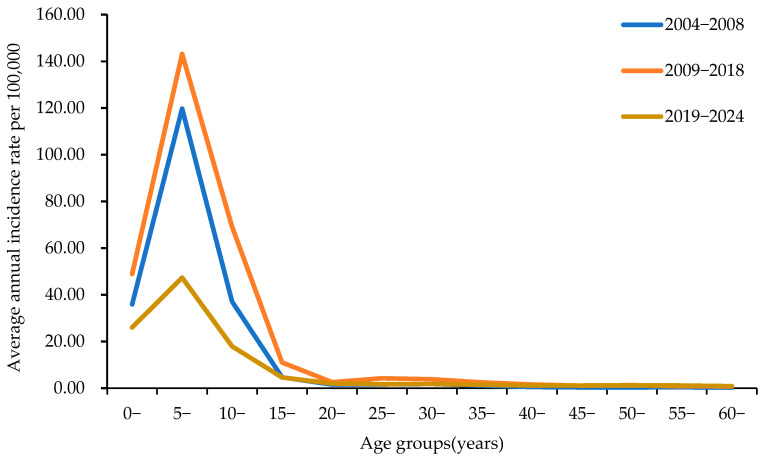
Age distribution of mumps incidence in Henan Province, 2004–2024.

Among all the patients, 58.11% were aged 5–9 years and were in kindergarten or primary school. Mumps incidence in Henan Province rose abruptly beginning at age 3, peaked at age 6, and then declined. The highest age-specific incidence occurred among children aged 5–9 years. Incidence fell rapidly in the 10–15-year group and continued to decline in subsequent age groups (≥15-years) (Figure 2).

**Figure 3:**
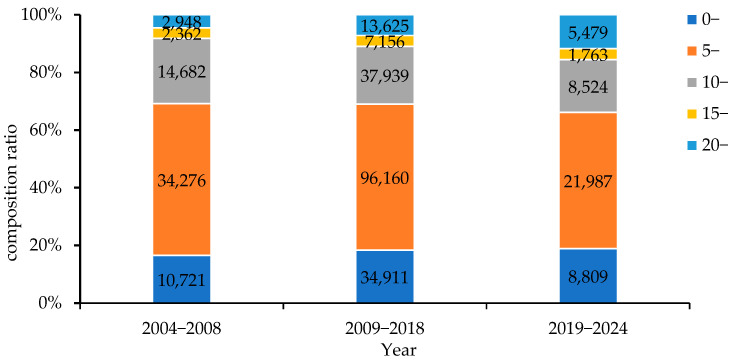
Age composition ratio of mumps cases in Henan Province, 2004–2024.

The proportion of children under 20 years old during the period of two doses of MuCV decreased significantly compared with that during the period of one dose (Figure 3). The percentage of the population over 20 years old tended to increase annually, from 8.17% in 2004–2008 to 15.55% in 2019–2024.

During the period of the two-dose MuCV immunization strategy, the reported incidence rates in the ≤4, 5–9, and 10–14 age groups decreased by 48.91% (χ^2^ = 634.252, *p* < 0.001), 64.35% (χ^2^ = 6017.069, *p* < 0.05), and 67.15% (χ^2^ = 656.884, *p* < 0.05), respectively (Table 3). Among the ten age groups of children under 10 years old, the differences in the decline in the reported incidence rates in all age groups were statistically significant. Among them, the decline rate of children in the 6-year-old group was the fastest, reaching 69.78%, while that of the 1-year-old group was the slowest.

### 3.6. Occupational Distribution

The main population affected by the disease is students (176,294 cases), followed by nursery-school children in daycare centres (62,526 cases) and pre-nursery children (38,627 cases), accounting for 58.50%, 20.75%, and 12.82% of the total number of cases, respectively. The proportions of students, nursery-school children, and pre-nursery children changed from 62.18%, 17.75%, and 13.95% from 2004–2008 to 54.36%, 25.48%, and 8.25% from 2019–2024, respectively (Table 4).

### 3.7. Estimation of MuCV Coverage

**Figure 4:**
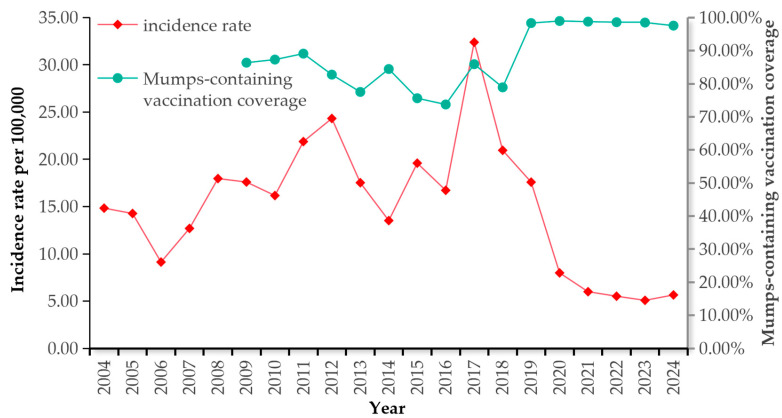
Evaluation of the relationship between vaccine vaccination coverage and mumps incidence.

There was an overall negative correlation between the estimated vaccination rate of MuCV and the reported incidence of mumps (r = −0.685, *p* < 0.05). During the period of the 1-dose MuCV immunization strategy, the estimated annual vaccination rate of MuCV fluctuated around 80%, with an average estimated vaccination rate of 82.15%. The reported incidence of mumps fluctuated around 20 per 100,000, with an epidemic cycle of 3–5 years, and peaked in 2017. During the period of the two-dose MuCV immunization strategy, the estimated annual vaccination rate of MuCV was maintained above 95% each year, with an average estimated vaccination rate of 98.45%. The reported incidence of mumps tended to decrease annually (χ^2^ = 42,469.246, *p* < 0.05). The reported incidence of mumps had an increasing trend in 2024, with a slight decrease in the vaccination rate (Figure 4).

## 4. Discussion

In 1990, mumps was designated a Class C notifiable infectious disease in China [10]. In 2004, the China CDC launched the National Notifiable Disease Reporting System (NNDRS), which began collecting nationwide mumps case counts [5]. Until 2008, the mumps vaccine was not included in China’s national immunization program, so parents paid for it out of pocket [7]. Consequently, the vaccine coverage rate was not high, and the incidence of mumps fluctuated at a relatively high level in Henan Province. After the 1-dose MMR program was launched, the incidence of mumps did not decrease considerably in Henan Province. Other studies have also shown that the effect of 1-dose MMR vaccination on controlling mumps is not ideal [11,12,13,14]. Over time, changes in human immunity to mumps reveal a complex scenario involving antibody attrition, seronegative conversion, and asymptomatic infections [15]. The antibody level against mumps has tended to decrease annually [16,17,18], sometimes even turning negative, which increases the number of susceptible individuals each year. The ongoing presence of asymptomatic carriers hinders the complete elimination of the infection source. Furthermore, the vaccination rate of a single-dose mumps vaccine (MuCV) has remained low at 82.15%, falling short of the threshold needed to achieve herd immunity. Consequently, the threshold for interrupting mumps transmission has not been met, and herd immunity has not been established. As a result, the mumps epidemic in Henan Province has recurred periodically [19,20], with an epidemic peak occurring every 3–5 years, consistent with the situation in the whole country [5]. With the reporting and management of mumps becoming more standardized, owing to the increased reporting sensitivity of mumps [21] and the limited protective effect of a single dose of MuCV [15,22], the mumps epidemic in Henan Province reached an epidemic peak in 2017. The F genotype of the mumps virus is the predominant wild-type strain circulating within China [23]. In contrast, China’s MMR program uses the S79 vaccine strain, derived from the American Jeryl-Lynn strain, classified under the A genotype. Research indicates that genetic mismatches can diminish cross-neutralization capacity [24,25,26]. Factors contributing to mumps outbreaks include the virus’s genetic evolution, genotype mismatches between vaccine and wild-type strains, and reduced antigenic cross-reactivity among viral genotypes [27,28,29,30,31]. This may also explain the high incidence of epidemic mumps in Henan Province during the period when the one-dose MuCV immunization strategy was implemented. However, conclusive data supporting this hypothesis is still lacking, and further molecular epidemiological research is needed.

In 2019, with the implementation of the two-dose MuCV immunization strategy, the MuCV vaccination rate in Henan Province increased significantly, with an average annual vaccination rate of 94.90%. The reported incidence of mumps in Henan Province did not show a periodic increase but rather a decreasing trend annually. In 2024, the reported incidence decreased by 73.03% compared with that in 2018 under the one-dose MuCV immunization strategy. This finding may be attributed to two causes: first, the 2019 implementation of the two-dose MuCV immunization strategy, combined with the high vaccination coverage (>90%), increased population immunity. The World Health Organization (WHO) recommends sustained, high immunization coverage and more than one vaccine dose for mumps prevention. The herd immunity threshold required to block mumps transmission has been estimated ≥86% [20]. Second, during the COVID-19 pandemic, targeted protective measures were implemented to block the transmission of respiratory diseases (including mumps). The implementation of these measures led to varying degrees of decline in the reported incidence rates of diseases with similar transmission routes to mumps after the COVID-19 pandemic [32].

There is a clear seasonal pattern of mumps incidence, with a sizeable annual peak between December and January and a small peak from April to July during the preimmunization planning period and the period of the 1-dose MuCV immunization strategy, which is generally consistent with the findings of previous studies [5,10,33]. The mumps virus is cold tolerant and can maintain its viability for 2 months at 4 °C. The suitable temperature in winter and spring in Henan accelerates the reproduction of mumps [10,34]. This finding is consistent with the situation in the whole country [5] and other regions [10,33,34] and conforms to the epidemic pattern of a high incidence of infectious respiratory diseases in winter and spring. Moreover, this high-incidence period coincides with the start of the school year. Given that the incubation period of mumps ranges from 12 to 28 days, upon students’ return to school, mumps commences to disseminate within the population. Subsequently, cases emerge after roughly one incubation period, which aligns with the aforementioned peak in the number of new cases. This phenomenon elucidates the relatively higher incidence of mumps among school-age children. The 2 doses of MuCV vaccination significantly weakened the seasonality of mumps in Henan Province; the incidence of epidemic mumps showed a sporadic distribution throughout the year, with no obvious seasonality.

In terms of regional distribution, mumps cases were reported in all regions of the province from 2004 to 2024. The high-incidence counties and districts showed dynamic changes during the preimmunization program period and the 1-dose and 2-dose MuCV immunization strategy periods. However, in all three periods, the high-incidence areas were mainly concentrated in the interprovincial border regions, the provincial capital, and its surrounding cities. This finding is consistent with previous research results [5], and conforms to the research results in other domestic regions [35]. In actuality, the urban environment thus furnishes a realistic backdrop for viral spread. In metropolitan areas, the high population density and the extensive public transportation systems in large cities act as a “multiplier effect” for the spread of viruses [36]. These areas have a large population flow and dense population, providing suitable conditions for the transmission of mumps virus. Moreover, in areas with a large floating population, the management of the floating population is difficult. The immunization program for these populations is relatively weak, and the vaccine coverage rate is relatively low. A critical vaccination level exists in the population such that if the degree of vaccine coverage is below the critical level, an epidemic will occur; otherwise, an epidemic will be prevented [37]. Therefore, the immune barrier of the population is not strong, and outbreaks and epidemics of the disease are prone to occur.

The age-specific incidence of mumps among children aged 5–9 years was the highest in Henan Province. In China, these age groups included children and adolescents attending kindergarten (3–6 years) and primary school (7–12 years). During the period of the two-dose MuCV immunization strategy, the reported incidence rates in all age groups among the population under 10 years old significantly decreased. Interestingly, the reported incidence rate of the 6-year-old group decreased the fastest, whereas that of the 1-year-old group decreased the slowest. There are two reasonable hypotheses for the abnormal phenomenon in which the decline in the 1-year-old group is slower than that in the other age groups. First, infants in the 0-year-old group have specific antibodies against mumps virus obtained from their mothers and are not easily infected. However, the level of maternally transmitted antibodies gradually decreases as the infants grow older, which may explain why children in the 1-year-old group are more susceptible to infection. Some studies have reported that the seropositive rate of mumps IgG is 40.0% in infants under 6 months old and 3.8% in infants aged 6–11 months [38]. Second, the slowest decline in the 1-year-old group may also be related to the fact that some children in this group received only one dose of MuCV and would not be fully protected. The 6-year-old group was the cohort population at the initial stage of the implementation of the two-dose MuCV immunization strategy and the age group with the highest incidence rate of mumps, indicating that implementing the two-dose MuCV vaccination strategy with a high vaccination rate among school-age children played a key role in reducing the incidence of mumps. These findings also suggest that, after two-dose MuCV vaccination, the protective effect of antibodies is relatively good in the short term, which is consistent with the results of other studies [39,40]. The waning immunity of mumps vaccines with different vaccine strains, such as Jeryl Lynn, Leningrad Zagreb, and Urabe, has been observed in other studies [13,38,41,42,43]. Therefore, further research is needed on the long-term persistence of antibody levels after two-dose MuCV vaccination.

With respect to sex, the reported incidence rate was higher in males than in females, which might be related to factors such as relatively lower levels of mumps antibodies in males, wider social activities, and poorer hygiene habits [35]. Besides the characteristic painful parotitis in infected males, orchitis represents the most prevalent extrasalivary manifestation [44]. Recent mumps resurgences have largely affected adolescents and young adults, with high incidences of orchitis being frequently documented [3,19,39]. Orchitis stands as the most common complication of mumps, occurring in up to 40% of all mumps cases among young adult males [3]. Mumps orchitis is predominantly unilateral, yet it can present bilaterally in 10–30% of cases [3]. Unvaccinated postpubertal males remain particularly susceptible to outbreaks and at high risk for mumps orchitis. Thus, males seem to have a greater risk of developing complications.

In terms of occupational distribution, students, nursery-school children and nurseries, and pre-nursery children were the main groups affected. However, after the implementation of the two-dose MuCV immunization strategy, the total composition ratio of this group decreased, which was consistent with the significant increase in the age distribution of people over 20 years old. Considering the high incidence of mumps in men, it is essential to be aware of this epidemiological shift.

The mumps vaccine is an effective means of preventing mumps. This study revealed that there was an overall negative correlation between the MuCV vaccination rate and the reported incidence of mumps, which was consistent with the results of other studies [6,39]. These findings also indirectly confirmed that mumps can be effectively controlled through vaccination. However, several mumps outbreaks among vaccinated young adults have recently been reported [24,39]. Antibody levels to the mumps vaccine component have been reported to decline more rapidly than antibody levels to the measles and rubella vaccine components [20]. This finding suggests the need for an additional dose of the vaccine during adolescence [45,46].

In conclusion, compared with the one-dose MuCV immunization strategy, the two-dose MuCV immunization strategy can effectively control the prevalence and outbreak of mumps—it significantly weakens the periodicity and seasonality of mumps incidence and greatly reduces the incidence rate in the immunized population. The proportion of the population aged over 20 years has increased. In the future, while continuing to implement a two-dose MuCV immunization strategy, consideration can be given to utilizing a third dose of MuCV among adolescents to address the changes in the epidemiological characteristics of mumps. Moreover, from an epidemiological perspective, the short-term protective effect of mumps antibodies after two-dose MuCV vaccination is relatively long-lasting. However, long-term observation and research are needed to evaluate the long-term immune persistence of mumps antibody levels and the long-term prevention and control of mumps after the implementation of the two-dose MuCV immunization strategy.

There are some limitations to this study. The data mainly originated from the direct reports of various medical institutions. The data of reported cases were directly influenced by the diagnosis and treatment levels of doctors in these institutions and their awareness of infectious disease reporting; thus, there might be under-reporting or misreporting. Moreover, the prevention and control measures implemented during the COVID-19 pandemic reduced the incidence of respiratory infectious diseases, including mumps, which would, to some extent, affect the evaluation of the prevention and control effectiveness of the two-dose immunization strategy.

## Figures and Tables

**Table 1:** The incidence rates and vaccination schedule of MuCV in Henan Province, China, 2004–2024.

Period	Case	Average Incidence (per 100,000)	95% CI	Vaccination Schedule
2004–2008	64,989	13.78	(9.77, 17.79)	Non-EPI
2009–2018	189,791	20.08	(16.25, 23.88)	1-dose MuCV included in EPI
2019–2024	46,562	7.93	(2.91, 13.02)	2-dose MuCV included in EPI

95% confidence intervals (95% CI): incidence rate of mumps.

**Table 2:** The incidence rates of mumps by district in Henan Province, China, 2004–2024.

District	Urbanization Rate(%)	Non-EPI			1-Dose MuCV Included in EPI			2-Dose MuCV Included in EPI		
		Case	Average Incidence (per 100,000)	95% CI	Case	Average Incidence (per 100,000)	95% CI	Case	Average Incidence (per 100,000)	95% CI
Zhengzhou	81.00	9120	26.54	(16.51, 36.57)	24,862	29.11	(23.43, 34.79)	8661	12.67	(4.98, 20.36)
Kaifeng	55.73	5144	21.9	(7.36, 36.43)	9337	20.06	(15.25, 24.87)	3138	11.27	(2.39, 20.15)
Luoyang	68.39	9126	28.58	(9.06, 48.11)	15,699	23.77	(15.36, 32.18)	4592	10.92	(7.56, 14.27)
Pingdingshan	57.24	3580	14.51	(8.60, 20.42)	13,112	26.44	(20.28, 32.59)	1613	5.39	(3.42, 7.36)
Anyang	56.86	6448	24.12	(10.77, 37.48)	15,036	28.63	(21.50, 35.76)	3787	11.95	(1.74, 22.15)
Hebi	64.34	2152	29.86	(6.95, 52.77)	3039	19.35	(14.59, 24.11)	1087	11.25	(−2.22, 24.73)
Xinxiang	61.15	2261	8.09	(4.73, 11.46)	8606	15.08	(2.74, 27.41)	1085	3.02	(1.59, 4.44)
Jiaozuo	66.22	1876	10.68	(2.66, 18.70)	4688	13.31	(3.85, 22.77)	2302	10.79	(4.02, 17.55)
Puyang	53.67	2610	14.62	(4.57, 24.67)	8476	23.08	(17.44 28.73)	2056	9.37	(0.12, 18.63)
Xuchang	57.26	1084	5.07	(1.83, 8.32)	7100	16.56	(8.42, 24.69)	1464	5.53	(3.19, 7.87)
Luohe	58.73	1591	13.58	(2.39, 24.76)	4093	16.13	(10.02, 22.23)	1094	7.29	(4.92, 9.66)
Sanmenxia	60.69	965	8.66	(1.22, 16.10)	3328	14.81	(7.94, 21.67)	1815	13.86	(−1.23, 28.94)
Nanyang	54.17	5768	12.06	(8.70, 15.42)	13,440	13.28	(10.91, 15.64)	2490	4.27	(1.77, 6.76)
Shangqiu	49.93	3472	8.05	(3.62, 12.48)	16,829	22.64	(16.31, 28.97)	3704	8.11	(0.94, 15.29)
Xinyang	53.85	2350	7.07	(−2.50, 16.64)	14,895	23.65	(14.41, 32.88)	2149	5.69	(3.93, 7.45)
Zhoukou	46.51	5094	10.05	(2.67, 17.43)	13,074	14.33	(10.37, 18.30)	2381	4.54	(1.29, 7.79)
Zhumadian	48.03	1883	4.88	(0.93, 8.83)	11,876	16.59	(10.42, 22.77)	1974	4.73	(2.76, 6.69)
Jiyuan	70.00	424	13.03	(5.78, 20.27)	2268	31.7	(−24.68, 88.08)	1170	26.83	(−0.98,54.64)

95% confidence intervals (95% CI): incidence rate of mumps.

**Table 3:** Epidemiological situation and changing trends of mumps among children aged < 10 years, in Henan Province, 2004–2024.

Age Groups (Year)	Average Incidence (per 100,000)			1-Dose Included in EPI			2-Dose Included in EPI		
	2004–2008	2009–2018	2019–2024	Average Decrease Rate (%)	χ^2^	*p*-Value	Average Decrease Rate (%)	χ^2^	*p*-Value
0–	2.95	4.44	2.35	−52.83	25.614	0.00	43.5	16.638	0.00
1–	6.40	12.41	8.17	−90.21	134.415	0.00	24.01	6.758	0.01
2–	19.77	32.61	18.14	−58.73	206.038	0.00	37.12	93.577	0.00
3–	54.62	78.68	38.16	−40.17	281.652	0.00	46.95	526.527	0.00
4–	98.22	123.69	50.51	−27.16	237.555	0.00	55.48	1398.989	0.00
5–	119.72	155.71	50.97	−29.48	336.154	0.00	64.46	2881.58	0.00
6–	140.41	185.36	50.21	−35.29	522.758	0.00	69.78	4733.896	0.00
7–	129.83	159.44	51.06	−20.51	199.088	0.00	64.59	3300.473	0.00
8–	125.78	122.11	46.39	2.79	4.166	0.04	61.49	2321.756	0.00
9–	87.28	103.81	38.66	−18.14	108.129	0.00	58.21	1579.612	0.00

*p*-value of χ^2^ test: the average of incidence compared with the previous period.

**Table 4:** Occupational distribution of reported mumps cases in Henan Province, 2004–2024.

Occupational Distribution	2004–2008			2009–2018			2019–2024		
	Cases	Composition Ratio (%)	95% CI	Cases	Composition Ratio (%)	95% CI	Cases	Composition Ratio (%)	95% CI
nursery-school children	11,533	17.75	(15.51, 19.99)	39,130	20.62	(19.55, 22.04)	11,863	25.48	(22.24, 33.52)
pre-nursery children	9069	13.95	(12.14, 15.53)	25,716	13.55	(11.48, 16.67)	3842	8.25	(7.01, 10.31)
Student	40,819	62.81	(58.54, 67.25)	110,166	58.05	(54.05, 60.78)	25,309	54.36	(42.20, 58.88)
Farmer	1932	2.97	(2.12, 3.89)	9579	5.05	(4.18, 5.81)	3488	7.49	(6.53, 9.73)
Others	1636	2.52	(2.15, 2.85)	5200	2.74	(2.49, 2.94)	2060	4.42	(3.85, 5.73)
Total	64,989	100	-	189,791	100	-	46,562	100	-

95% confidence intervals (95% CIs): composition ratio.

## Data Availability

The original contributions presented in this study are included in the article. Further inquiries can be directed to the corresponding author.

## References

[1] Xu J., Wang Y., Duan G., Liu F., Yang H. (2023). Impact of non-pharmaceutical interventions during COVID-19 pandemic on measles and mumps in Mainland China. J. Infect..

[2] Rana M.S., Usman M., Alam M.M., Tahir M., Ikram A., Zaidi S.S.Z., Kashif M., Umair M., Anas M., Ullah N. (2023). The emergence of mumps after the COVID-19 pandemic in Pakistan: Time to consider MMR vaccination strategies. J. Infect..

[3] Wu H., Wang F., Tang D., Han D. (2021). Mumps Orchitis: Clinical Aspects and Mechanisms. Front. Immunol..

[4] Takagi A., Ohfuji S., Nakano T., Kumihashi H., Kano M., Tanaka T. (2022). Incidence of Mumps Deafness in Japan, 2005–2017: Analysis of Japanese Insurance Claims Database. J. Epidemiol..

[5] Fu X., Ge M., Xu W., Yu M., Ju J., Zhong Y., Huang H. (2022). Epidemiological features and sociodemographic factors associated with mumps in mainland China from 2004 to 2018. J. Med. Virol..

[6] Zhang Y., Lu M., Mahesh K.C., Kim E., Shamseldin M.M., Ye C., Dravid P., Chamblee M., Park J.G., Hall J.M. (2022). A highly efficacious live attenuated mumps virus-based SARS-CoV-2 vaccine candidate expressing a six-proline stabilized prefusion spike. Proc. Natl. Acad. Sci. USA.

[7] Shi C., Liu W.H., Yang L., Yan Z.L., Li L., Zhang Z.B., Ou C.Q. (2022). A Multi-Age-Group Interrupted Time-Series Study for Evaluating the Effectiveness of National Expanded Program on Immunization on Mumps. Vaccines.

[8] Yin Z., Wen T., Fang Q., Zheng C., Gong X., Li J., Wang S., Xiang Z. (2022). Assessment of mumps-containing vaccine effectiveness by dose during 2006 to 2020 in Quzhou, China. Hum. Vaccines Immunother..

[9] Orenstein W.A., Bernier R.H., Dondero T.J., Hinman A.R., Marks J.S., Bart K.J., Sirotkin B. (1985). Field evaluation of vaccine efficacy. Bull. World Health Organ..

[10] Huang J.F., Zhao Z.Y., Lu W.K., Rui J., Deng B., Liu W.K., Yang T.L., Li Z.Y., Li P.H., Liu C. (2022). Correlation between mumps and meteorological factors in Xiamen City, China: A modelling study. Infect. Dis. Model..

[11] Sun X., Tang F., Hu Y., Deng X., Wang Z., Zhou M., Liu Y. (2020). High risk of mumps infection in children who received one dose of mumps-containing vaccine: Waning immunity to mumps in children aged 2–5 years from kindergartens in Jiangsu Province, China. Hum. Vaccines Immunother..

[12] Ma C., Liu Y., Tang J., Jia H., Qin W., Su Y., Wang H., Hao L. (2018). Assessment of mumps-containing vaccine effectiveness during an outbreak: Importance to introduce the 2-dose schedule for China. Hum. Vaccines Immunother..

[13] Liu Y., Liu Z., Deng X., Hu Y., Wang Z., Lu P., Guo H., Sun X., Xu Y., Tang F. (2018). Waning immunity of one-dose measles-mumps-rubella vaccine to mumps in children from kindergarten to early school age: A prospective study. Expert Rev. Vaccines.

[14] Jiang R.J., Yin Q.Z., Xu M.J., Zhao Z.M., Deng Y., Che Y.C. (2019). Epidemiological characteristics of mumps in mainland China from 2004 to 2018 and key population for prevention and control. Chin. J. Contemp. Pediatr..

[15] Liu Y., Xiong Y., Liang Y., Deng X., Hu Y., Hu R., Chen Q., Tang F., Wang Z., Sun X. (2021). Waning immunity and potential asymptomatic infection in 3–7 years old children who received one dose of measles-mumps-rubella vaccine: A 4-year prospective study. Vaccine.

[16] Singh A.K., Phatak S.R., Singh R., Bhattacharjee K., Singh N.K., Gupta A., Sharma A. (2022). Humoral antibody kinetics with ChAdOx1-nCOV (Covishield™) and BBV-152 (Covaxin™) vaccine among Indian Healthcare workers: A 6-month longitudinal cross-sectional Coronavirus Vaccine-induced antibody titre (COVAT) study. Diabetes Metab. Syndr..

[17] Levin E.G., Lustig Y., Cohen C., Fluss R., Indenbaum V., Amit S., Doolman R., Asraf K., Mendelson E., Ziv A. (2021). Waning Immune Humoral Response to BNT162b2 COVID-19 Vaccine over 6 Months. N. Engl. J. Med..

[18] Cohen C., White J.M., Savage E.J., Glynn J.R., Choi Y., Andrews N., Brown D., Ramsay M.E. (2007). Vaccine effectiveness estimates, 2004–2005 mumps outbreak, England. Emerg. Infect. Dis..

[19] Su S.B., Chang H.L., Chen A.K. (2020). Current Status of Mumps Virus Infection: Epidemiology, Pathogenesis, and Vaccine. Int. J. Environ. Res. Public Health.

[20] Kaaijk P., Wijmenga-Monsuur A.J., Ten Hulscher H.I., Kerkhof J., Smits G., Nicolaie M.A., van Houten M.A., van Binnendijk R.S. (2022). Antibody Levels at 3-Years Follow-Up of a Third Dose of Measles-Mumps-Rubella Vaccine in Young Adults. Vaccines.

[21] Choe Y.J., Lee Y.H., Cho S.I. (2017). Increasing mumps incidence rates among children and adolescents in the Republic of Korea: Age-period-cohort analysis. Int. J. Infect. Dis..

[22] Dong Y., Wang L., Burgner D.P., Miller J.E., Song Y., Ren X., Li Z., Xing Y., Ma J., Sawyer S.M. (2020). Infectious diseases in children and adolescents in China: Analysis of national surveillance data from 2008 to 2017. BMJ (Clin. Res. Ed.).

[23] Cui A., Zhu Z., Mao N., Si Y., Ma Y., Hu Y., Deng X., Wang L., Zeng L., Zhang Y. (2018). Assessment of one-dose mumps-containing vaccine effectiveness on wild-type genotype F mumps viruses circulating in mainland China. Vaccine.

[24] Vermeire T., Barbezange C., Francart A., Hamouda A., Litzroth A., Hutse V., Martens L., Vandermarliere E., Van Gucht S. (2019). Sera from different age cohorts in Belgium show limited cross-neutralization between the mumps vaccine and outbreak strains. Clin. Microbiol. Infect..

[25] Park S.H. (2015). Resurgence of mumps in Korea. Infect. Chemother..

[26] Rubin S.A., Qi L., Audet S.A., Sullivan B., Carbone K.M., Bellini W.J., Rota P.A., Sirota L., Beeler J. (2008). Antibody induced by immunization with the Jeryl Lynn mumps vaccine strain effectively neutralizes a heterologous wild-type mumps virus associated with a large outbreak. J. Infect. Dis..

[27] Gouma S., Koopmans M.P., van Binnendijk R.S. (2016). Mumps virus pathogenesis: Insights and knowledge gaps. Hum. Vaccines Immunother..

[28] Gonçalves G., Frade J., Nascimento M.S., Mesquita J.R., Nunes C. (2016). Persistence of rubella and mumps antibodies, following changes in the recommended age for the second dose of MMR vaccine in Portugal. Epidemiol. Infect..

[29] Liu Y., Hu Y., Deng X., Wang Z., Lu P., Ma F., Zhou M., Liu P., Min J. (2015). Seroepidemiology of mumps in the general population of Jiangsu province, China after introduction of a one-dose measles-mumps-rubella vaccine. Sci. Rep..

[30] Hukic M., Hajdarpasic A., Ravlija J., Ler Z., Baljic R., Dedeic Ljubovic A., Moro A., Salimović-Besic I., Sausy A., Muller C.P. (2014). Mumps outbreak in the Federation of Bosnia and Herzegovina with large cohorts of susceptibles and genetically diverse strains of genotype G, Bosnia and Herzegovina, December 2010 to September 2012. Eurosurveillance.

[31] Fiebelkorn A.P., Lawler J., Curns A.T., Brandeburg C., Wallace G.S. (2013). Mumps postexposure prophylaxis with a third dose of measles-mumps-rubella vaccine, Orange County, New York, USA. Emerg. Infect. Dis..

[32] Zhang Z., Liu N., Zhang J., Xu J., Wang W., Xiao J., Wang T., Luan L., Zhang Y. (2022). Epidemiological Characteristics of Varicella under Different Immunisation Strategies in Suzhou Prefecture, Jiangsu Province. Vaccines.

[33] Zhang Q., Zhou M., Yang Y., You E., Wu J., Zhang W., Jin J., Huang F. (2019). Short-term effects of extreme meteorological factors on childhood hand, foot, and mouth disease reinfection in Hefei, China: A distributed lag non-linear analysis. Sci. Total Environ..

[34] Zhang H., Su K., Zhong X. (2022). Association between Meteorological Factors and Mumps and Models for Prediction in Chongqing, China. Int. J. Environ. Res. Public Health.

[35] Pang H., Zhou Y., Zhao W., Jiang Q. (2021). Epidemiological changes in mumps infections between 1990 and 2017 in urban area of Shanghai, China. Hum. Vaccines Immunother..

[36] Guida C., Carpentieri G. (2021). Quality of life in the urban environment and primary health services for the elderly during the COVID-19 pandemic: An application to the city of Milan (Italy). Cities.

[37] Cai C.R., Wu Z.X., Guan J.Y. (2014). Effect of vaccination strategies on the dynamic behavior of epidemic spreading and vaccine coverage. Chaos Solitons Fractals.

[38] Bassal R., Shohat T., Levin T., Pando R., Shinar E., Amichay D., Barak M., Ben-Dor A., Bar-Haim A., Mendelson E. (2022). The Concordance between Mumps and Rubella Sero-Positivity among the Israeli Population in 2015. Vaccines.

[39] Wang D., Nie T., Pan F., Wang Y., Wang J., Qin W. (2021). Loss of protective immunity of two-dose mumps-containing vaccine over time: Concerns with the new strategy of the mumps immunization program in China. Hum. Vaccines Immunother..

[40] Kaaijk P., Emmelot M.E., Kerkhof J., van Els C., Meiring H.D., de Wit J., Bodewes R. (2021). Genetic Analysis Reveals Differences in CD8(+) T Cell Epitope Regions That May Impact Cross-Reactivity of Vaccine-Induced T Cells against Wild-Type Mumps Viruses. Vaccines.

[41] Rota J.S., Rosen J.B., Doll M.K., McNall R.J., McGrew M., Williams N., Lopareva E.N., Barskey A.E., Punsalang A., Rota P.A. (2013). Comparison of the sensitivity of laboratory diagnostic methods from a well-characterized outbreak of mumps in New York city in 2009. Clin. Vaccine Immunol..

[42] Pang H., Zhou Y., Zhao W., Jiang Q. (2018). Seroprevalence and Determinants Associated with Mumps Antibodies after 20 Years of MMR Vaccination in Urban Area of Shanghai, China. Int. J. Environ. Res. Public Health.

[43] Lewnard J.A., Grad Y.H. (2018). Vaccine waning and mumps re-emergence in the United States. Sci. Transl. Med..

[44] Masarani M., Wazait H., Dinneen M. (2006). Mumps orchitis. J. R. Soc. Med..

[45] Zhang X., Sridharan S., Zagoriy I., Eugster Oegema C., Ching C., Pflaesterer T., Fung H.K.H., Becher I., Poser I., Müller C.W. (2023). Molecular mechanisms of stress-induced reactivation in mumps virus condensates. Cell.

[46] Cardemil C.V., Dahl R.M., James L., Wannemuehler K., Gary H.E., Shah M., Marin M., Riley J., Feikin D.R., Patel M. (2017). Effectiveness of a Third Dose of MMR Vaccine for Mumps Outbreak Control. N. Engl. J. Med..

